# Effects of theta burst stimulation on the Parkinsonian gait disorder and cortical gait-network activity

**DOI:** 10.1177/1877718X251320941

**Published:** 2025-05-18

**Authors:** Janina Dutke, Jonas Gehlenborg, Miriam Heise, Wolfgang Hamel, Christian Gerloff, Götz Thomalla, Tim Magnus, Andreas K Engel, Christian KE Moll, Alessandro Gulberti, Monika Pötter-Nerger

**Affiliations:** 1 Department of Neurology, University Medical Center Hamburg-Eppendorf, Hamburg, Germany; 2 Department of Neurosurgery, University Medical Center Hamburg-Eppendorf, Hamburg, Germany; 3 Department of Neurophysiology and Pathophysiology, University Medical Center Hamburg-Eppendorf, Hamburg, Germany

**Keywords:** Parkinson’s disease, gait, deep brain stimulation, subthalamic nucleus, theta burst stimulation, electroencephalography

## Abstract

**Background:**

The Parkinsonian gait disorder and freezing of gait (FoG) are challenging symptoms of Parkinson's disease (PD).

**Objective:**

To assess the effect of subthalamic theta burst deep brain stimulation (TBS-DBS) on the Parkinsonian gait performance in real-world conditions and cortical activity indexed by mobile EEG.

**Methods:**

In this monocentric, randomised, double-blind, short-term study, 12 age-matched controls (11 male, age 59 ± 8 years) and 15 PD participants (14 male, age 62 ± 9 years, disease duration 15 ± 6 years) with subthalamic stimulation (76 ± 39 months) were assessed with clinical scores (FoG-Course, MDS-UPDRS) and a standardized gait course simulating everyday life situations. Three DBS algorithms were applied in a randomized order with intertrial waiting periods of 30 min: (1) OFF-DBS; (2) cDBS; (3) TBS-DBS (interburst frequency 5 Hz, intraburst frequency 200 Hz) with regular medication. During the standardized gait course a mobile, 24-channel EEG system and 6 wearable axial kinematic sensors were used.

**Results:**

The primary outcome, the relative change of FoG-Course by DBS, was not superior with TBS-DBS compared to cDBS in the entire sample. Seven of fifteen PD participants rated subjectively TBS-DBS equal or better than cDBS (“TBS-preference group”). EEG recordings revealed movement-induced alpha and beta suppression in premotor and motor cortex in both cDBS and TBS-DBS conditions in PD with slightly different patterns between the DBS modes.

**Conclusions:**

In this pilot trial, TBS-DBS showed benefits in the subjective perception of gait in a subgroup of PD patients accompanied by specific cortical network changes. TBS-DBS merits further investigation in future larger cohort studies with longer observation periods.

## Introduction

The Parkinsonian gait disorder is one of the most challenging symptoms of Parkinson's disease (PD), with a considerable reduction in quality of life.^1^ Freezing of gait (FoG) is a particularly disabling form of gait impairment, which is often experienced during step initiation, turning, passing narrow points, or distraction.^2^

Treatment options for alleviating the Parkinsonian gait disorder include deep brain stimulation of the subthalamic nucleus (STN-DBS). Conventional STN-DBS (cDBS) with 130 Hz and 60 μs pulse width has been demonstrated to improve most motor symptoms including some aspects of the Parkinsonian gait disorder, such as levodopa-responsive FoG.^3^ While there is a constant, long-term cDBS effect on tremor and rigidity, there is a loss of cDBS efficacy on the Parkinsonian gait disorder and FoG in the postoperative course of 5 years after surgery.^4^ One possible explanation for this outcome is the disease progression involving non-dopaminergic systems, which might not be impacted by cDBS. Another hypothesis could be that gait disturbance and FoG are caused by suboptimal DBS programming or even by effects of DBS-induced maladaptive neuroplasticity.^5^ In PD patients with chronic STN-DBS, FoG was shown to be reversible by reprogramming DBS parameters as low-frequency stimulation.^6^ Consequently, stimulation-refractory or even stimulation-induced gait disturbances require the development of new stimulation algorithms.

Previously, a new type of DBS, the so-called theta burst stimulation (TBS-DBS) has been transferred from non-invasive transcranial magnetic stimulation^7^ and has been evaluated to be safe in PD patients.^7,8^ TBS-DBS is characterized by temporally grouped stimulation pulses with an inter-burst frequency of 5 Hz and better symptomatic efficacy at higher intra-burst frequency around 200 Hz. Clinical effects of unilateral TBS-DBS on contralateral PD motor symptoms have already been demonstrated,^7^ but there is no knowledge about the impact of bilateral TBS-DBS on the Parkinsonian gait and the associated cortical network activities.

The aims of the present, single-center, randomized, double-blind study were to (1) assess the effects of bilateral TBS-DBS on gait and FoG in PD patients by using established clinical scores, quantitative gait analysis with wearable kinematic sensors in real life gait conditions and (2) cortical network changes measured by a mobile EEG system. We hypothesized that 1. bilateral TBS-DBS might be superior to cDBS in improving the Parkinsonian gait disorders reflected by improved clinical measures of gait and freezing of gait 2. this clinical improvement is associated with concurrent, distinct, cortical network changes.

## Methods

The study was conducted in agreement with the Code of Ethics of the World Medical Association (declaration of Helsinki, 1967) and was approved by the local ethics committee (Ethik-Kommission der Ärztekammer Hamburg, PV6025). All PD participants and control subjects gave their written informed consent to participate.

### Participants

Inclusion criteria were (1) PD participants with presence of a relevant Parkinsonian gait disorder with at least 1 point in the MDS-UPDRS item 3.10 and 2 points in the postural instability and gait disorder (PIGD) sum score (MDS-UPDRS items 2.12–2.13, 3.10–3.12)^9^ (note that it was not explicitly mandatory to suffer from FoG)^10^; (2) bilateral STN-DBS therapy in a stable treatment condition >3 months postoperatively; (3) implanted Medtronic Activa system enabling the patterned stimulation mode. Exclusion criteria were (1) other than PD associated gait-affecting neurological or musculoskeletal impairments; (2) PD participants unable to walk. All PD participants were tested with their normal dopaminergic medication to simulate daily life conditions. 12 healthy controls matched in sex and age (11 male, 1 female, age 59 ± 8 years) to the PD participants were also recorded.

### Protocol and experimental setup

The double-blinded, clinical assessments included (1) a freezing of gait course (FoG-Course), which is a standardized rating instrument for FoG. The FoG-Course includes four different maneuvers as standing up from a chair, start walking for 1 m, turning 360° clockwise and counter-clockwise, opening and walking through a door. For each maneuver, the rater assigns a score from 0 to 3 points: 0 points, if the patient did not show any festination or FoG; 1 point for festination or hastening; 2 points for FoG that the patient overcomes independently; 3 points if the patient needs assistance from the examiner to overcome the FoG episode. This course was repeated i) without any additional task (“walking”), ii) with a simultaneous, second motor task by carrying a tray with a cup of water (“carrying”), and iii) with additional motor and mental task performing a serial mathematical calculation (“carrying and calculation”).^11^ (2) the Movement Disorder Society – Unified Parkinson's Disease Rating Scale III (MDS-UPDRS III)^12^ and (3) a standardized 115-meter walking course through the floors of the local neurology department. This course featured daily life walking challenges such as passing doors (6 times), using an elevator, turning (4 times 90° and once 180°) and climbing stairs. Most of these daily life walking conditions are known to provoke FoG.^2^ In this pilot trial, the PD participants performed these tasks in randomized order and an intertrial interval of >30 min in three stimulation conditions: (1) OFF-DBS; (2) conventional STN-DBS (130 Hz); and (3) high-frequency TBS-DBS (interburst-frequency 5 Hz and intraburst-frequency 200 Hz) with TEED (total electrical energy delivered) adjusted amplitudes. In detail, the TBS pattern consists of 5 cycles per second, with each cycle containing 0.1s bursts at 200 Hz followed by a 0.1s pause. The experimental cDBS parameters differed from the clinical parameters prior to the experimental session in some PD participants, as we aimed to consistently use a clinically effective, monopolar, omnidirectional stimulation mode at a single ring contact for the experimental cDBS condition.

To characterise the PD participants in detail, all PD participants performed during their usual therapeutic stimulation clinical and neurocognitive tests as the Montreal Cognitive Assessment (MoCA),^13^ the Movement Disorder Society – Unified Parkinson's Disease Rating Scale I, II and IV (MDS-UPDRS),^12^ the Giladi FoG-Q,^10^ the Parkinson's disease questionnaire (PDQ-39)^14–16^ and the Beck's depression inventory (BDI-II),^17,18^ which are used in the clinical routine.

### Kinematic recordings and analysis

During the standardized walking course, all PD participants and healthy controls wore 6 kinematic sensors (ADPM, INC., Portland, OR) with integrated triaxial accelerometer, magnetometer, and gyroscope attached at both ankles and wrists, at the lower back and at the chest. Data were collected (sampling frequency 128 Hz), extracted off-line with the APDM software Motion Studio (APDM, INC., Portland, OR) and imported into Matlab R2020a (Mathworks, Natick, MA) for further analysis. First, left and right heelstrikes were marked as events using an algorithm to detect local minima in acceleration curves. In a second step, these heelstrikes were used to analyse stepping frequency (steps per second), mean step duration (seconds), step duration of better and worse side (seconds), coefficient of variation of step duration, stepping asymmetry (step duration better/worse side), and total stepping time (seconds) in the straight walking sections. Especially, the coefficient of variation of step duration and stepping asymmetry are associated with FoG.^19,20^ In a third step, FoG episodes were defined as episodes with a stepping frequency within the “freeze” band (3–8 Hz) in comparison to the locomotion band (0.5–3 Hz).^21^ To detect episodes of akinetic freezing, which could not be distinguished from voluntary stopping, freezing analyses were corrected by visual inspection, written protocols and video recordings from the experiment. Based on these detection algorithms, the total number of FoG episodes and the total and relative freezing time (total freezing time (sec)/total stepping time (sec) as ratio) were calculated.

To investigate the observation that some PD participants benefitted from the TBS-DBS while others did not, PD participants were classified into two groups: TBS-preference group and Non-TBS-preference group. In order to focus primarily on the individual preference from the patient's perspective, PD participants were asked to rank the three conditions explicitly in terms of their gait disturbance and FoG while still blinded to DBS condition. On this basis, the TBS-preference group was defined as PD participants who subjectively rated the TBS-DBS condition as better (4 PD participants) or equal (3 PD participants) to the cDBS.

### EEG recordings and analysis

For EEG recordings, the portable EEG system Smarting Mobi (mbraintrain, Belgrade, RS), with 24-channel EEG cap with Ag/AgCl sintered ring electrodes by EasyCap (EASYCAP GmbH, Herrsching, Germany) was used. EEG signals were recorded with the accompanying smartphone app (sampling rate 500 Hz, impedances <10 kOhm). Further analyses were performed with Matlab R2020a (Mathworks, Natick, MA) and the open-source Matlab toolboxes EEGLAB2019_1^22^ and FieldTrip*.*^23^ For synchronization with kinematic data, the implemented gyroscope of the EEG system was used. EEG data were referenced to average, high-pass filtered (Kaiser-windowed FIR filter with a cutoff frequency of 0.75 Hz, bandwidth 0.5 Hz, stopband attenuation −80 dB) to reduce slow drifts and low-pass filtered (Kaiser-windowed FIR filter with a cutoff frequency at 95 Hz, bandwidth 10 Hz, stoppband attenuation −80 dB) to eliminate high-frequency DBS artifacts. DBSFilt toolbox^24^ was applied to eliminate artifacts in harmonics and subharmonics of the stimulation frequency. Line noise artifacts of 50 Hz were reduced by the Cleanline plugin^25^ for EEGLAB. The Clean Rawdata plugin for EEGLAB removed flat line channels and reconstructed short-lasting irregular artifacts as, e.g., muscle artifacts by artifact subspace reconstruction (ASR).^26^ Data portions with a variance larger than 12 SD relative to calibration data were removed, validated by visual inspection. Furthermore, adaptive mixture of independent component analysis (AMICA)^27^ was applied and components representing more than 85% muscle, heart or eye artifacts as well as channel or line noise, classified by ICLabel^28^ were removed. Additionally, DIPFIT plugin for EEGLAB by Robert Oostenveld was used to remove components with dipoles located outside the head and components with residual variance over 20%.

As we aimed to analyze FFT Spectra in different conditions, the EEG data were segmented into 1 s non-overlapping epochs^29^ to compare episodes of straight walking, FoG and resting-like condition (30s EEG recording while the patient is sitting on a chair with eyes open before starting the FoG-Course) among participants. To standardize and equally weight each participant in the grand average, power spectra were calculated for each 1 s epoch and averaged on subject level between 2 and 45 Hz with a frequency resolution of 1 Hz and a frequency smoothing of ± 2 Hz with Slepian sequence multitaper. For spectral power analysis, the open-source Fieldtrip toolbox^23^ was used. For analysis of spectral power distributions in changes between motor states (resting-like vs. walking and walking vs. freezing), power spectra were calculated for each DBS condition. These absolute power spectra were first transformed to natural logarithmic power and then depicted as line plots. The main frequency bands were defined as delta (1–3 Hz), theta (4–8 Hz), alpha (9–13), low-beta (14–20), high-beta (21–30), and gamma (31–45 Hz). Due to muscle artifacts during walking, we did not consider the EEG results in the gamma band. Line plots were shown for two different regions of interest (ROIs), with central ROI representing motor execution in motor cortex (Cz, C3, C4) and frontal ROI representing motor planning in premotor cortex (Fz, F3, F4).

### Statistical analysis

Statistical analyses were performed with SPSS Statistics 26 (IBM Corp., New York, USA). As most variables did not show a normal distribution, non-parametric statistical procedures were preferred. Because 4 PD participants were not able to perform the entire clinical assessment in at least one condition (2 in the OFF-DBS condition, 1 in both the OFF-DBS and TBS-DBS conditions and 1 in the TBS-DBS condition) due to severe symptom load and exhaustion, specific parts of the analysis were conducted with a subset of 11 PD participants for whom complete data sets were available. The primary outcome was the difference between stimulation-induced relative changes of the FoG-Course by cDBS and TBS-DBS.

### Behavioral data

The primary outcome, the relative difference of FoG-Course between cDBS and TBS-DBS was evaluated by Wilcoxon signed-rank test.

Healthy controls (HCs) and PD participants were compared by multivariate analysis of variance (MANOVA) and consecutive, explorative univariate analyses of variance. Within the PD group, comparisons between the three stimulation conditions were performed by non-parametric Friedman ANOVAs with Dunn-Bonferroni post-hoc tests.

We investigated whether the PD participants’ subjective clinical overall impression of their TBS-DBS preferences was reflected in the clinical scores and objective gait parameters by calculating a biserial correlation.

We examined potential predictive factors to define TBS-preference group by using a binary-logistic regression with the variables disease duration, MDS-UPDRS II, PDQ-39 and LEDD. The choice of possible predictors for the linear model was based on scatter plots and theoretical assumptions.

### EEG analysis

We used the non-parametric Monte Carlo cluster-based permutation test, implemented in the FieldTrip toolbox,^23^ to investigate relative power changes over the whole cortex. This test reduces error susceptibility through multiple comparisons without relying on a-priori assumptions concerning the distribution. For each frequency band and channel, a distribution of t-values for comparison between resting-like and walking in every DBS condition and for comparison between TBS-preference group and Non-TBS-preference group was built. Significant clusters were defined as two or more neighboring channels with a p-value below 0.05. For a high accuracy, the number of random permutations was set to 5000.

## Results

15 PD participants (14 male, 1 female, age 62 ± 9 years, disease duration 15 ± 6 years) and subthalamic stimulation (STN-DBS for 76 ± 39 months) participated in this study (Table 1). All enrolled PD participants suffered from typical clinical characteristics of the Parkinsonian gait disorder (Table 2) and all reported experiencing FoG as indicated in the freezing of gait questionnaire after Giladi (FoG-Q).^10^

**Table 1.** Clinical and demographic characteristics of PD participants.

											Clinical DBS parameters	cDBS parameters	TBS-DBS parameters
Sex	Disease duration	Time with DBS	PIGD-Score	LEDD	MoCA	BDI-II	PDQ-39	FoG-Q	FoG - COURSE	MDS-UPDRS-III	left electrode	left electrode	left electrode
Age	[years]	[months]		[mg]					OFF/cDBS/TBS	OFF/cDBS/TBS	right electrode	right electrode	right electrode
M 51*	12	13	10	817	25	2	13.12	25	18/19/21	24/13/24	3.5 mA, 130 Hz, 60μs, E2-C+	3.5 V, 130 Hz, 60μs, E2-C+	4.0 V, 200 Hz, 60μs, E2-C+
											3.0 mA, 130 Hz, 60μs, E10-C+	3.0 V, 130 Hz, 60μs, E10-C+	3.4 V, 200 Hz, 60μs, E10-C+
M 70	14	64	3	829	28	7	11.82	12	21/7/3	34/18/20	3.5 V, 125 Hz, 60μs, E0-C + / 3.5 V, 125 Hz, 60μs, E1-C+ 3.5 V, 125 Hz, 60μs, E8-C + / 3.5 V, 125 Hz, 60μs, E9-C+	3.5 V, 130 Hz, 60μs, E1-C+ 3.5 V, 130 Hz, 60μs, E9-C+	4.0 V, 200 Hz, 60μs, E1-C+ 4.0 V, 200 Hz, 60μs, E9-C+
M 57	13	109	7	1583	23	17	51.82	28	4/6/12	31/30/30	1.3 V, 125 Hz, 60μs, E1-C + / 0.8 V, 125 Hz, 60μs, E2-C + 3.6 V, 125 Hz, 60μs, E9-C + / 3.6 V, 125 Hz, 60μs, E10-C+	2.5 V, 130 Hz, 60μs, E2-C+ 3.4 V, 130 Hz, 60μs, E10-C+	2.9 V, 200 Hz, 60μs, E2-C+ 3.0 V, 200 Hz, 60μs, E10-C+
M 68	12	37	4	1240	26	9	38.70	27	29/2/7	26/29/25	1.5 V, 90 Hz, 60μs, E1-E2+ 2.0 V, 90 Hz, 60μs, E10-E11+	1.0 V, 130 Hz, 60μs, E1-C+ 1.3 V, 130 Hz, 60μs, E10-C+	1.2 V, 200 Hz, 60μs, E1-C+ 1.5 V, 200 Hz, 60μs, E10-C+
M 61	11	43	2	1169	26	8	18.80	17	3/3/1	27/26/28	3.1 V, 130 Hz, 60μs, E2-C+ 2.8 V, 130 Hz, 60μs, E10-C+	3.1 V, 130 Hz, 60μs, E2-C+ 2.8 V, 130 Hz, 60μs, E10-C+	3.5 V, 200 Hz, 60μs, E2-C+ 3.2 V, 200 Hz, 60μs, E10-C+
M 60	18	76	12	680	26	14	36.09	20	22/19/22	61/29/46	3.6 V, 160 Hz, 60μs, E3-C+ 3.5 V, 160 Hz, 60μs, E10-C+	3.9 V, 130 Hz, 60μs, E3-C+ 3.8 V, 130 Hz, 60μs, E10-C+	4.4 V, 200 Hz, 60μs, E3-C+ 4.3 V, 200 Hz, 60μs, E10-C+
M56	8	24	4	804	25	11	30.26	7	14/9/12	48/22/29	2.8 V, 130 Hz, 60μs, E2-C+ 2.5 V, 130 Hz, 60μs, E9-C+	2.8 V, 130 Hz, 60μs, E2-C+ 2.5 V, 130 Hz, 60μs, E9-C+	3.2 V, 200 Hz, 60μs, E2-C+ 2.9 V, 200 Hz, 60μs, E9-C+
M 70*	16	59	11	475	16	8	24.95	21	36/2/0	77/37/34	3.5 mA, 125 Hz, 60μs, E1-C + / 1.5 mA, 125 Hz, 60μs, E3-C+ 3.0 mA, 125 Hz, 60μs, E9-C + / 1.8 mA, 125 Hz, 60μs, E11-C+	3.0 V, 130 Hz, 60μs, E2-C+ 3.1 V, 130 Hz, 60μs, E10-C+	3.4 V, 200 Hz, 60μs, E2-C+ 3.5 V, 200 Hz, 60μs, E10-C+
M 39	12	96	9	389	21	48	37.60	26	0/0/0	47/26/21	3.4 mA, 130 Hz, 60μs, E1-C+ 3.6 mA 130 Hz, 60μs, E9-C+	3.4 V, 130 Hz, 60μs, E1-C+ 3.6 V, 130 Hz, 60μs, E9-C+	3.9 V, 200 Hz, 60μs, E1-C+ 4.1 V, 200 Hz, 60μs, E9-C+
M 70	18	90	9	1230	24	11	40.52	15	7/5/5	37/28/27	3.0 mA, 130 Hz, 60 μs, E3-C+ 2,5 mA, 130 Hz, 60μs, E11-C+	2.5 V, 130 Hz, 60μs, E2-C+ 2.2 V, 130 Hz, 60μs, E10-C+	1.6 V, 200 Hz, 60μs, E2-C+ 2.0 V, 200 Hz, 60μs, E10-C+
M 72	8	61	6	540	23	1	17.24	29	17/9/14	42/20/27	3.0 V, 130 Hz, 60μs, E1-C+ 1.2 V, 130 Hz, 60μs, E10-C+	3.0 V, 130 Hz, 60μs, E1-C+ 1.2 V, 130 Hz, 60μs, E10-C+	2.7 V, 200 Hz, 60μs, E1-C+ 1.4 V, 200 Hz, 60μs, E10-C+
W 65*	22	139	10	537	24	10	35.73	35	27/11/24	61/31/43	2.0 V, 160 Hz, 60μs, E0-E3+ 2.0 V, 160 Hz, 60μs, E9-E11+	2.2 V, 130 Hz, 60μs, E1-C+ 1.6 V, 130 Hz, 60μs, E9-C+	2.5 V, 200 Hz, 60μs, E1-C+ 1.8 V, 200 Hz, 60μs, E9-C+
M 61	15	101	6	731	27	8	39.48	42	22/1/6	53/40/35	1.9 V, 90 Hz, 60μs, E3-C+ 4.2 V, 90 Hz, 60μs, E11-C+	1.7 V, 130 Hz, 60μs, E1-C+ 2.0 V, 130 Hz, 60μs, E10-C+	1.9 V, 200 Hz, 60μs, E1-C 2.2 V, 200 Hz, 60μs, E10-C++
M 68	29	149	9	1348	26	3	54.06	37	2/0/28	46/48/45	4.3 V, 130 Hz, 60μs, E1-C+ 3.7 V, 130 Hz, 60μs, E10-C+	4.3 V, 130 Hz, 60μs, E1-C+ 3.7 V, 130 Hz, 60μs, E10-C+	4.9 V, 200 Hz, 60μs, E1-C+ 4.2 V, 200 Hz, 60μs, E10-C+
M 61*	21	84	18	1897	27	10	46.51	34	1/1/11	84/25/74	2.5 V, 125 Hz, 60μs, E2-C + / 1.6 V, 125 Hz, 60μs, E3-C+ 2.7 V, 125 Hz, 60μs, E10-C + / 0.9 V, 125 Hz, 60μs, E11-C+	2.7 V, 130 Hz, 60μs, E2-C+ 3.0 V, 130 Hz, 60μs, E10-C+	3.1 V, 200 Hz, 60μs, E2-C+ 3.4 V, 200 Hz, 60μs, E10-C+
Mean ± SD	15 ± 6	76 ± 39	8 ± 4	951 ± 441	24 ± 3	11 ± 11	33 ± 13	25 ± 10/34 ± 12	15 ± 11/6 ± 6/11 ± 9	47 ± 18/28 ± 19/34 ± 14			
Median; IQR	14; 6	76; 48	9; 5	817; 625	25; 3	9; 4	36; 18	26; 13/38; 17	17; 19/5; 8/11; 14	46; 25/28; 7/29; 13			

Demographic data from individual participants (upper rows) and mean/median data are shown. “Disease duration [years]” is calculated from the date of diagnosis to the date of baseline measurement. “Postoperative time [months]” is calculated from the date of surgery to the date of the baseline testing. “PIGD-Score” is the mean of MDS-UPDRS items 2.12, 2.13, 3.10, 3.11 and 3.12 in OFF-DBS. PIGD: postural instability/gait difficulty; LEDD: Levodopa equivalent daily dose; MoCA: Montreal Cognitive Assessment score; PDQ-39: Parkinsońs Disease Questionnaire; BDI- II: Becks Depression Inventory; MDS-UPDRS: Unified Parkinsońs Disease Rating Scale of the Movement Disorder Society; OFF: OFF-DBS; cDBS: conventional STN-DBS; TBS: theta burst stimulation; FoG-Course: freezing of gait course; E: electrode contact; C: cage; *Participant was removed from the analysis of objective gait parameters because of incapability to complete the standardized walking course in all three DBS conditions. FoG-Q: Giladi Freezing-Questionnaire.

**Table 2.** Gait characteristics of PD participants and healthy controls (HCs) in the standardized 115 m walking course.

					HCs vs. PD participants				DBS conditions		
	HCs	OFF-DBS	cDBS	TBS-DBS	HCs vs. OFF	HCs vs. cDBS	HCs vs. TBS	Friedman ANOVA	OFF vs. cDBS	OFF vs. TBS	cDBS vs. TBS
asymmetry ratio	1.0197 ± 0.0157,	1.0853 ± 0.0560,	1.0776 ± 0.0831,	1.0627 ± 0.0545,	*F* = 16.213	*F* = 6.512	*F* = 7.305	χ^2^ = 3.455			
	1.0151; 0.0202	1.0813; 0.0663	1.0410; 0.1032	1.0424; 0.0388	*p* = 0.001	*p* = 0.017	*p* = 0.012	*p* = 0.178			
stepping frequency [steps/s]	1.7088 ± 0.0980,	1.8307 ± 0.2227,	1.8789 ± 0.1464,	1.9599 ± 0.2214,	*F* = 1.295	*F* = 9.426	*F* = 5.603	χ^2^ = 4.545			
	1.7166; 0.1322	1.8298; 0.3484	1.9117; 0.0926	1.9792; 0.1636	*p* = 0.267	*p* = 0.005	*p* = 0.026	*p* = 0.103			
step duration [s]	0.5829 ± 0.0340,	0.5513 ± 0.0661,	0.5314 ± 0.0480,	0.5154 ± 0.0634,	*F* = 0.477	*F* = 8.086	*F* = 1.752	χ^2^ = 4.545			
	0.5799; 0.0438	0.5416; 0.1008	0.5223; 0.0280	0.5018; 0.0434	*p* = 0.497	*p* = 0.009	*p* = 0.198	*p* = 0.103			
CV of step duration	5.0866 ± 1.3043,	7.8785 ± 2.5781,	7.1058 ± 2.8222,	7.1936 ± 4.1132,	*F* = 12.574	*F* = 6.023	*F* = 1.938	χ^2^ = 3.455			
	5.1957; 1.1983	7.6660; 2.9611	6.5376; 3.4807	5.5270; 4.2941	*p* = 0.002	*p* = 0.021	*p* = 0.177	*p* = 0.178			
step duration better side [s]	0.5885 ± 0.0336,	0.5740 ± 0.0753,	0.5510 ± 0.0608,	0.5318 ± 0.0768,	*F* = 0.000	*F* = 2.448	*F* = 5.929	χ^2^ = 4.545			
	0.5891; 0.0374	0.5544; 0.1105	0.5328; 0.0274	0.5139; 0.0466	*p* = 0.998	*p* = 0.130	*p* = 0.023	*p* = 0.103			
step duration worse side [s]	0.5773 ± 0.0350,	0.5287 ± 0.0594,	0.5118 ± 0.0426,	0.4990 ± 0.0512,	*F* = 2.135	*F* = 17.046	*F* = 19.680	χ^2^ = 5.636			
	0.5742; 0.0500	0.5287; 0.0958	0.5118; 0.0313	0.4941; 0.0417	*p* = 0.158	*p* = 0.000	*p* = 0.000	*p* = 0.060			
relative freezing time [ratio]	0	0.0226 ± 0.0433,	0.0011 ± 0.0036,	0.0390 ± 0.1163,	*F* = 4.068	*F* = 1.999	*F* = 3.131	χ^2^ = 3.000			
		0.0000; 0.0140	0.0000; 0.0000	0.0000; 0.0085	*p* = 0.056	*p* = 0.170	*p* = 0.090	*p* = 0.223			
total freezing time [s]	0	4.1808 ± 7.9163,	0.1186 ± 0.3935,	4.9952 ± 14.8708,	*F* = 4.654	*F* = 1.662	*F* = 1.927	χ^2^ = 6.741	*z* = 0.864	*z* = 0.500	*z* = 0.364
		1.2800; 2.0085	0.0000; 0.0000	0.0000; 1.0900	*p* = 0.042	*p* = 0.209	*p* = 0.178	*p* = 0.034	*p* = 0.128	*p* = 0.723	*p* = 1.000
total freezing number	0	0.8636 ± 1.2266,	0.0909 ± 0.3015,	0.9545 ± 2.1030,	*F* = 7.470	*F* = 1.938	*F* = 3.553	χ^2^ = 5.120			
		0.5000; 1.000	0.0000; 0.0000	0.0000; 0.7500	*p* = 0.012	*p* = 0.176	*p* = 0.072	*p* = 0.077			
total stepping time [s]	102.3333 ± 10.5923,	138.8906 ± 33.9724,	113.2123 ± 7.8003,	118.7273 ± 10.9073,	*F* = 7.013	*F* = 8.650	*F* = 2.818	χ^2^ = 6.857	*z* = 1.091	*z* = 0.545	*z* = 0.545
	102.0000; 7.3750	131.5000; 42.2500	112.3350; 8.7500	124.5000; 18.7500	*p* = 0.015	*p* = 0.007	*p* = 0.106	*p* = 0.032	*p* = 0.032	*p* = 0.602	*p* = 0.602

HCs: healthy controls; OFF = OFF-DBS; cDBS: conventional STN-DBS; TBS: theta burst STN-DBS; CV: coefficient of variation. Values are given as mean ± SD (upper rows) and median; IQR (lower rows). MANOVA between HCs and PD participants revealed significant intersubject differences (HCs vs. OFF-DBS *p* = 0.004; HCs vs. cDBS *p* = 0.000; HCs vs. TBS-DBS *p* = 0.000). Explorative univariate analysis of variance are given in the table. For intrasubject comparison between DBS conditions Friedman ANOVAs were calculated. When ANOVA showed a significant difference, post hoc pairwise comparisons were performed.

### Behavioral results

During the standardized walking course, 7 participants showed FoG in the OFF-DBS condition, 2 participants in the cDBS condition, and 5 participants in the TBS-DBS condition. Four PD participants, who were not able to perform the course in all three stimulation conditions, were excluded from gait analyses.

#### The group effect of TBS-DBS is not superior to cDBS on clinical scores

The primary endpoint was defined as the difference in stimulation-induced changes of the FoG-Course across the different DBS algorithms compared to the OFF-DBS condition. The comparison of the relative DBS–induced change in the FoG-Course from OFF-DBS in the entire sample revealed slightly better effects of cDBS compared to TBS-DBS (Wilcoxon signed rank test *p* = 0.039). When the comparison was restricted to the subgroup of TBS-preference group, the FoG-Course was equally improved by both stimulation modalities.

**Figure 1.**
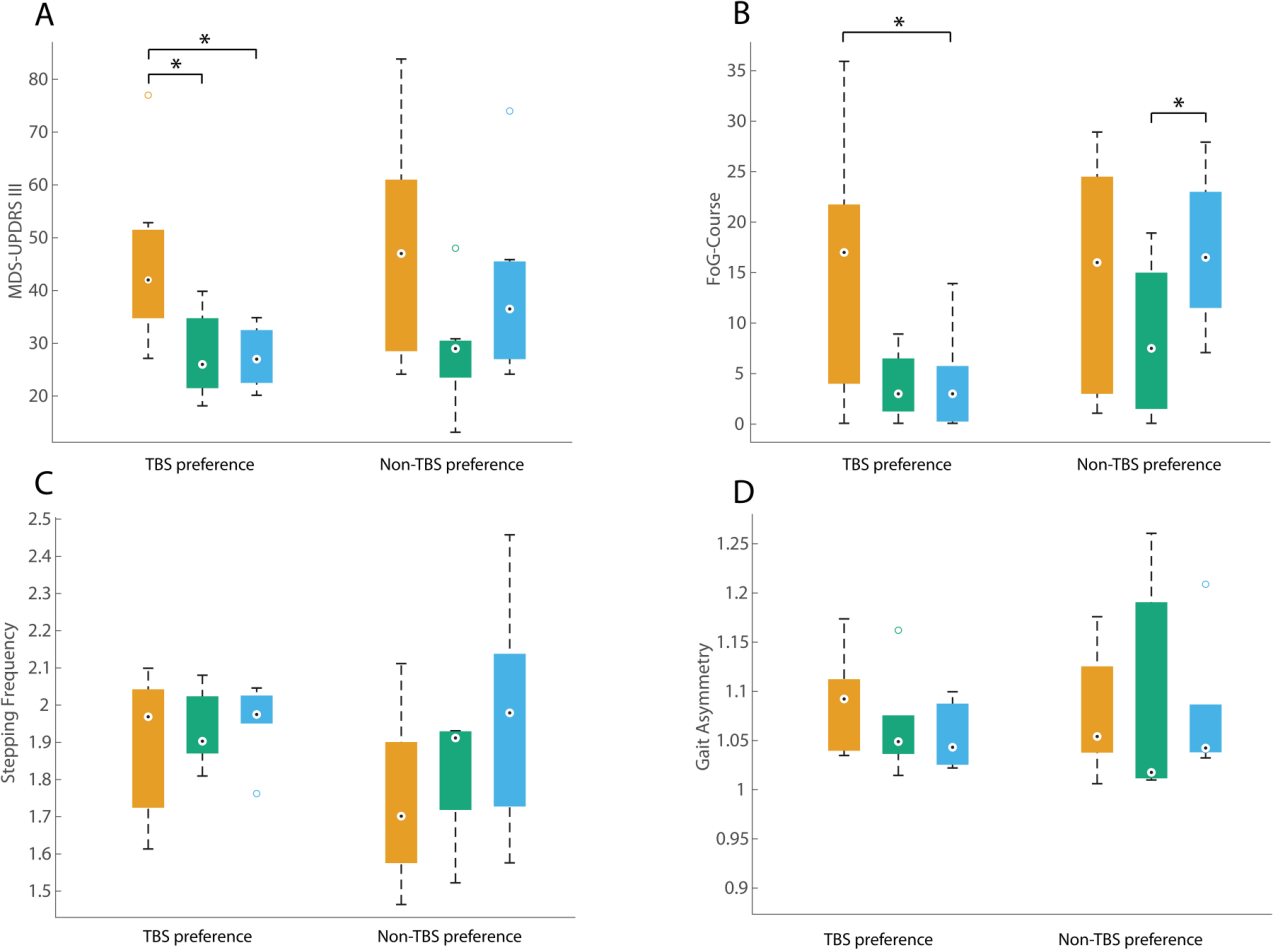
Comparison within the TBS preference and Non-TBS preference subgroups. Results of the MDS-UPDRS III (A), FoG-Course (B), and of the gait parameters stepping frequency (C) and gait asymmetry (D), across the three DBS conditions: OFF-DBS (orange, left box plots), cDBS (green, middle box plots), and TBS-DBS (blue, right box plots). The boxplots represent the 25th and 75th percentiles (box boundaries), while the whiskers indicate approximately 99.3% of the data (assuming a normal distribution). Outliers are marked as circles. Level of significance was set at 0.05.

The differential impact on the FoG-Course by the three DBS conditions (Friedmann ANOVA *χ^2^* = 7.654, *df* = 2, *p* = 0.022) revealed in pairwise comparisons a significant improvement between cDBS (*z* = 0.933, *p* = 0.032) vs. OFF-DBS. The FoG-Course was not significantly different between TBS-DBS and OFF-DBS and TBS-DBS and cDBS in the whole sample (all *p* values > 0.362. If the ANOVA was carried out only within the subgroup of TBS-preference group, the results were different; only TBS-DBS showed a significant improvement compared to OFF-DBS (*z* = 1.286, *p* = 0.048; Figure 1, Panel B).

General motor symptoms as indexed by the total score of the MDS-UPDRS III were differentially affected (Friedman ANOVA *χ^2^* = 13.759, *df* = 2, *p* = 0.001). Post-hoc pairwise comparisons revealed significant motor improvement by cDBS (*z* = 1.2, *p* = 0.003), as well as TBS-DBS (*z* = 1.1, *p* = 0.008) in comparison to OFF-DBS, whereas the comparison of cDBS and TBS-DBS showed no significant difference (*z* = -0.1, *p* = 1.000) (Table 1). The same results can be found, if the ANOVA was carried out only within the subgroup of the TBS-preference group. Both cDBS and TBS-DBS showed a significant improvement compared to OFF-DBS (cDBS vs. OFF-DBS *z* = 1.286, *p* = 0.048; TBS-DBS vs. OFF-DBS *z* = 1.286, *p* = 0.048; Figure 1, Panel A).

#### Neither DBS condition normalizes objective gait parameters

The comparison of the objective gait parameters, measured in the standardized 115 m walking course with everyday life walking challenges, between HCs and PD participants revealed significant differences in all three DBS conditions (MANOVA HC vs. OFF-DBS: Hotelling`s trace: 3.806, *p* = 0.004; HC vs. cDBS: Hotelling's trace = 4.224, *p* < 0.001; HC vs. TBS-DBS: Hotelling`s trace = 7.671, *p* < 0.001). As expected for most variables, significant differences were found between HCs and PD participants in all conditions. These findings indicate, that DBS was unable to normalize gait characteristics in PD to the level of the controls (Table 2).

#### Most objective gait parameters did not differ between the DBS conditions

Within the PD group, we tested whether there were effects of the DBS condition on the objective temporal and spatial gait parameters. The total stepping time was significantly improved by cDBS in comparison with OFF-DBS (Friedmann ANOVA *χ^2^* = 6.857, *p* = 0.0032; post hoc test *z* = 1.091, *p* = 0.032). No significant changes in gait velocity, gait asymmetry or CV of step duration were observed between DBS conditions. Also all other objective gait parameters did not show significant improvements (Table 2). Noticeably, there was a large variability among the PD participants.

#### The TBS-preference subgroup showed specific improvement of FoG

All 15 PD participants were asked to subjectively rate the TBS-DBS condition when still blinded. 4 PD participants rated TBS better (4 PD participants), equal (3 PD participants) or worse (8 PD participants) compared to cDBS. In 11 participants, complete results were available in all tested DBS conditions and were analysed. The TBS-preference group (*n* = 6) and Non-TBS-preference group (*n* = 5) were similarly affected in the OFF-DBS condition in terms of general motor symptom load (MDS-UPDRS III: *U* = 27.000, *p* = 0.955) and FoG severity (FoG-Course: *U* = 27.000, *p* = 0.955). In the cDBS condition, clinical scores showed similar improvements (MDS-UPDRS III: *U* = 25.000, *p* = 0.779; FoG-Course: *U* = 19.000, *p* = 0.336) in the TBS-preference group and Non-TBS-preference group. However, in the TBS-DBS condition, there was a significant group difference for the FoG-Course (MDS-UPDRS III: *U* = 14.000, *p* = 0.121; FoG-Course: *U* = 4.000, *p* = 0.004), indicating specific improvement of FoG irrespective of general motor symptom load by TBS-DBS in this subgroup. As the PD participants’ evaluation of TBS-DBS focused on their gait and gait irregularities, the clinical FoG-Course aligns effectively with the patient's subjective judgement. The biserial correlation between TBS-DBS preference and the clinical scores showed a high correlation between the TBS-DBS preference and the total score in the FoG-Course (*r* = -0.732, *p* = 0.002), the TBS-preference group showed lower scores in the FoG-Course than the Non-TBS-preference group. It should be noted that two PD participants from the Non-TBS-preference group were unable to complete the gait assessment in the TBS-DBS condition due to severe symptom load.

#### MDS-UPDRS II could be a potential predictor of the TBS preference

To assess potential predictive factors for identifying PD patients, who might become part of the TBS-preference group, a binary logistic regression was conducted with disease duration, total score of MDS-UPDRS II, total score of PDQ-39 and LEDD as factors. The analysis resulted in a significant linear model (*χ^2^* = 5.568, *p* = 0.018) with one potential predictor, namely, the MDS-UPDRS II (beta weight *p* = 0.092). Table 3 provides an overview of clinical characteristics of the TBS-preference group and Non-TBS-preference group, of note there were no significant differences of epidemiological factors or clinical scores in OFF-DBS or cDBS.

**Table 3.** Clinical comparison of PD TBS-preference group and Non-TBS-preference group.

	PD participants			TBS preference vs. Non-preference (Mann-Whitney-U-Test)	Healthy controls (*n* = 12)
	All (*n* = 15)	TBS preference (*n* = 7)	Non-TBS preference (*n* = 8)		
Age [years]	61.93 ± 8.83,	63.29 ± 11.63,	60.75 ± 6.04,	*U* = 41,000	59.00 ± 8.33
	61.00; 10.50	70.00; 9.00	60.50; 9.00	*p* = 0.152	
Disease duration [years]	15.27 ± 5.64,	13.43 ±3.36,	16.88 ± 6.90,	*U* = 20,000	n.a.
	14.00; 6.00	14.00; 4.00	15.50; 9.25	*p* = 0.397	
Postoperative time [months]	76.33 ± 39.29,	73.43 ± 22.07,	78.88 ± 51.52,	*U* = 27,000	n.a.
	76.00; 47.50	64.00; 33.00	80.00; 82.75	*p* = 0.955	
LEDD	951.10 ± 440.78,	766.06 ± 332.10,	1113.00 ± 479.42,	*U* = 15,000	n.a.
	816.50; 625.32	731.25; 491.61	1028.15; 633.33	*p* = 0.152	
MoCA	24.47 ± 2.97,	23.57 ± 4.12,	25.25 ± 1.28,	*U* = 23,000	28.42 ± 1.51
	25.00; 2.50	24.00; 4.50	25.50; 1.25	*p* = 0.613	
PDQ-39	33.11 ± 13.40,	27.20 ± 11.89,	38.29 ± 13.14,	*U* = 17,000	n.a.
	36.09; 18.13	24.95; 20.52	37.40; 13.48	*p* = 0.232	
BDI-II	11.13 ± 11.04,	13.00 ± 15.73,	9.50 ± 5.04,	*U* = 21,500	1.5 ± 2.11
	9.00; 3.50	8.00; 2.00	10.00; 4.25	*p* = 0.463	
FoG-Q	25.00 ± 9.79,	23.14 ± 10.25,	26.63 ± 9.75,	*U* = 21,000 / U = 18,000	n.a.
	26.00; 13.00/	21.00; 11.50/	27.50; 10.50/	*p* = 0.463 / p = 0.281	
	33.80 ± 11.50,	30.57 ± 10.42,	36.63 ± 12.32,		
	38.00; 17.00	30.00; 13.50	39.00; 11.25		
MDS-UPDRS Ia	3.27 ± 3.69,	4.43 ± 4.65,	2.25 ± 2.49,	*U* = 35,000	n.a.
	2.00; 5.50	2.00; 6.50	2.00; 3.00	*p* = 0.463	
MDS-UPDRS Ib	8.27 ± 6.72,	5.43 ± 4.83,	10.75 ± 7.44,	*U* = 17,500	n.a.
	7.00; 10.50	4.00; 4.00	11.00; 12.25	*p* = 0.232	
MDS-UPDRS II	17.07 ± 7.54,	12.86 ± 3.67,	20.75 ± 8.31,	*U* = 11,000	n.a.
	15.00; 7.00	14.00; 4.00	20.00; 11.00	*p* = 0.054	
MDS-UPDRS IV	4.27 ± 5.20,	4.29 ± 5.16,	4.25 ± 5.60,	*U* = 28,500	n.a.
	3.00; 6.00	3.00; 4.00	2.00; 7.00	*p* = 1.000	
MDS-UPDRS III					
OFF-DBS	46.53 ± 18.16,	45.29 ± 16.38,	47.63 ± 20.65,	*U* = 27,000	n.a.
	46.00; 24.50	42.00; 14.50	47.00; 31.25	*p* = 0.955	
cDBS	28.13 ± 8.79,	27.86 ± 8.13,	28.38 ± 9.88,	*U* = 25,000	n.a.
	28.00; 7.00	26.00; 9.50	29.00; 6.00	*p* = 0.779	
TBS-DBS	33.87 ± 13.80,	27.43 ± 5.74,	39.50 ± 16.59,	*U* = 14,000	n.a.
	29.00; 13.00	27.00; 7.00	36.50; 17.25	*p* = 0.121	
FoG-Course					
OFF-DBS	14.87 ± 11.49,	15.14 ± 12.67,	14.63 ± 11.24,	*U* = 27,500	n.a.
	17.00; 18.50	17.00; 16.50	16.00; 19.75	*p* = 0.955	
cDBS	6.27 ± 6.24,	3.86 ± 3.29,	8.38 ± 7.60,	*U* = 19,000	n.a.
	5.00; 7.50	3.00; 4.50	7.50; 11.25	*p* = 0.336	
TBS-DBS	11.07 ± 9.15,	4.14 ± 4.95,	17.13 ± 7.53,	*U* = 4000	n.a.
	11.00; 13.50	3.00; 5.00	16.50; 10.75	*p* = 0.004	

“Disease duration [years]” is calculated from the date of diagnosis to the date of baseline measurement. “Postoperative time [months]” is calculated from the date of surgery to the date of baseline testing. Values are given as mean ± SD (upper rows) and median; IQR (lower rows). LEDD: Levodopa equivalent daily dose; MoCA: Montreal Cognitive Assessment score; PDQ-39: Parkinsońs Disease Questionaire; BDI- II: Becks Depression Inventory; MDS-UPDRS: Unified Parkinsońs Disease Rating Scale of the Movement Disorder Society; FoG-Course: freezing of gait course; OFF: OFF-DBS; cDBS: conventional STN-DBS; TBS: theta-burst STN-DBS. FoG-Course in TBS-DBS condition showed a significant difference between the TBS preference and Non-TBS preference group. FoG-Q: Giladi Freezing-Questionnaire.

### EEG results

#### TBS-DBS and cDBS showed slightly different patterns of movement-induced alpha- and beta-suppression

**Figure 2.**
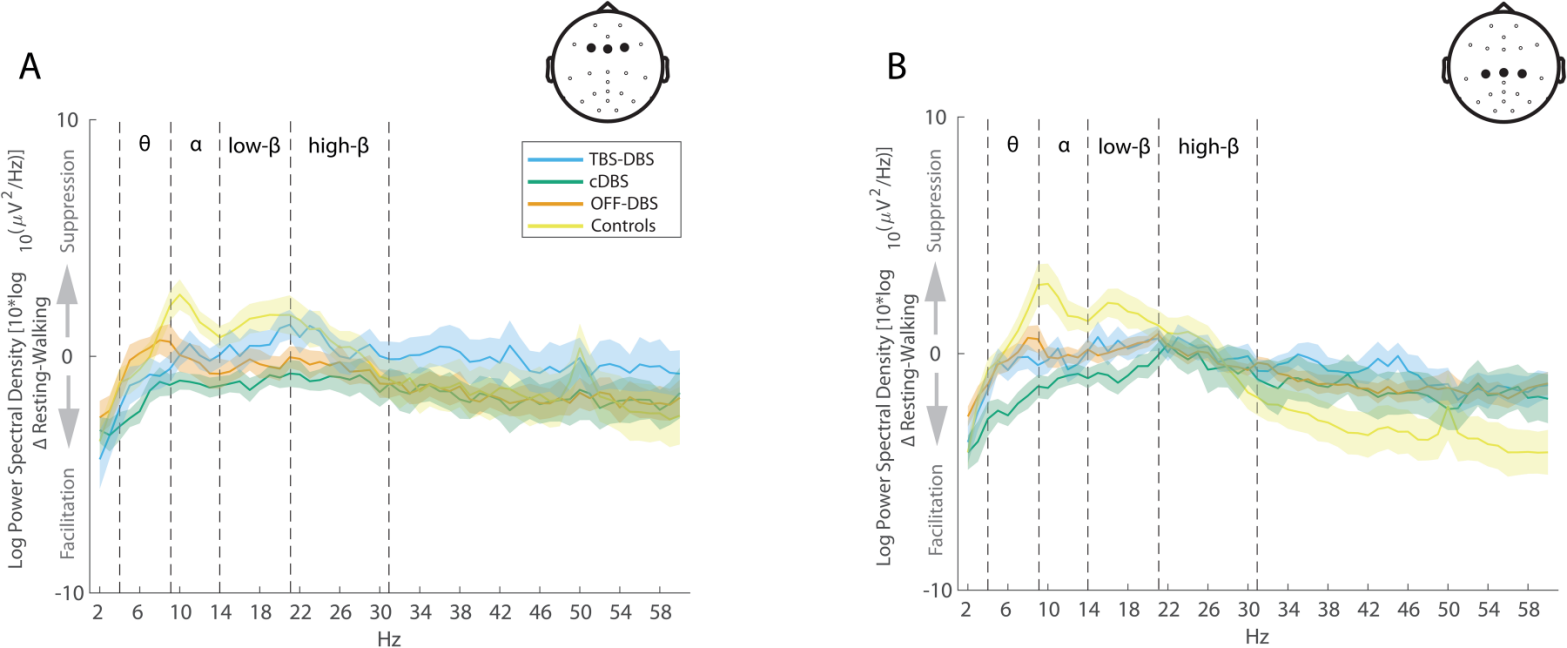
Relative movement-induced cortical activity changes in controls and PD participants during different DBS stimulation conditions during straight, regular gait without FoG episodes. Averaged cortical activity modulation (Δ Resting – Walking) is shown for the frontal ROI (Fz, F3 and F4 electrodes; panel A) and the central ROI (Cz, C3 and C4 electrodes; panel B) in controls (yellow) and PD participants in different stimulation conditions (OFF-DBS (orange), cDBS (green), TBS-DBS (blue)). The shaded area represents the standard error of the mean. Movement-induced alpha- and beta-suppression is less accentuated in PD participants compared to controls; TBS-DBS seems to be more effective than cDBS in restoring low-beta suppression, particularly in the frontal ROI in PD participants.

The DBS-induced clinical and gait changes described above were associated with specific cortical activity modulation indexed by EEG. In line with previous studies, there was a movement-induced suppression of alpha and beta cortical oscillatory activity in HCs during walking when compared to the “resting-like” state (Figure 2). In PD participants, this alpha and beta suppression was generally less pronounced in all DBS conditions compared to HCs. When comparing oscillatory activity changes in the frontal ROI across different DBS conditions, movement-induced beta suppression was more pronounced during TBS-DBS than during cDBS and OFF-DBS (Figure 2).

**Figure 3.**
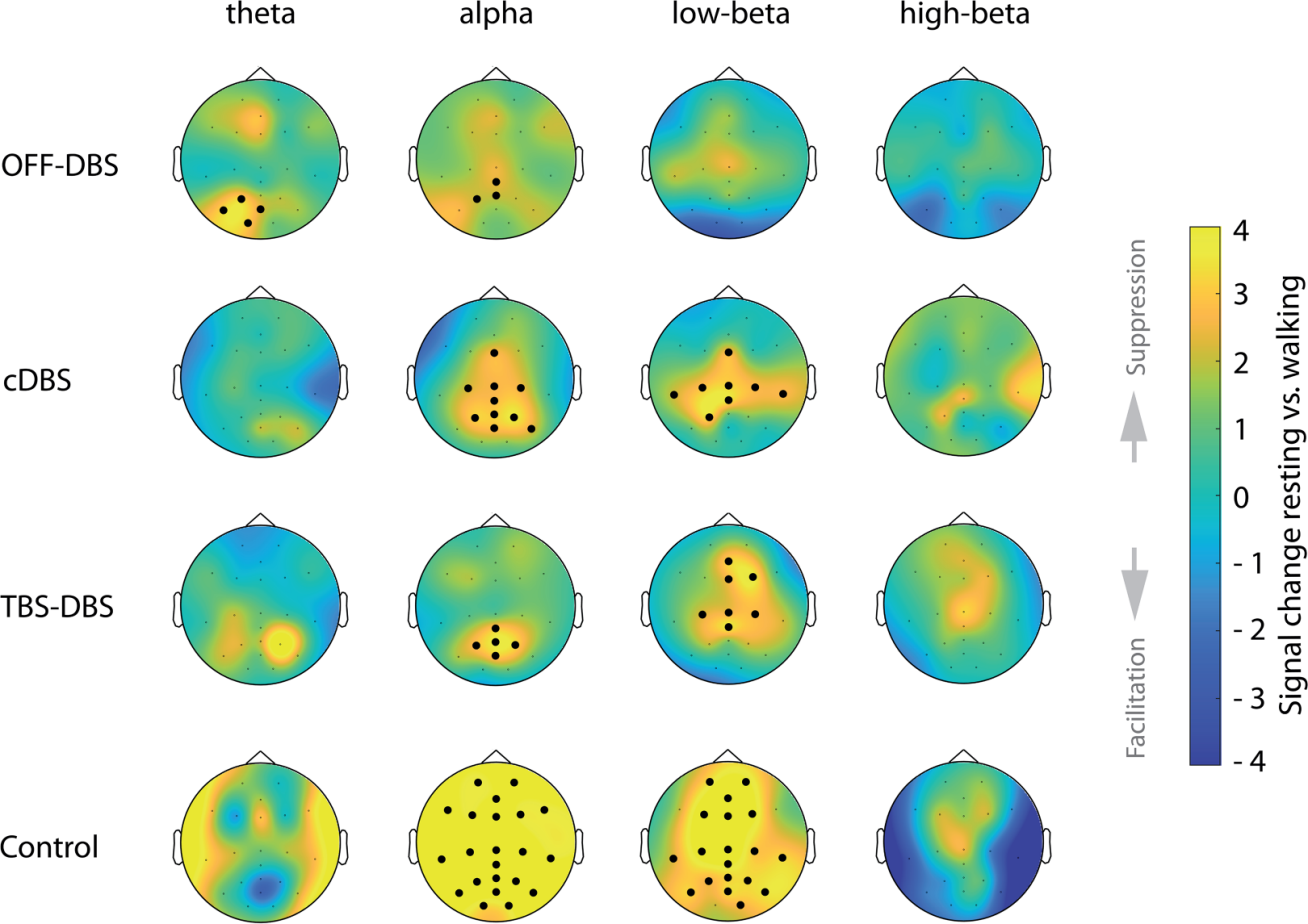
Topographical distribution of movement-induced cortical oscillatory activity changes. Cortical activity modulation, averaged over all PD participants, (topographic percentage power signal change, calculated with Montecarlo cluster permutation test) between “resting-like” and walking conditions for each PD participants in OFF-DBS (first row), cDBS (second row), TBS-DBS (third row) and healthy controls (lowest row). Significant clusters are indicated by bold dots. Note that the color scaling is adjusted to highlight the smaller EEG power changes in PD, which might be accompanied by exaggeration of color changes in controls. Movement-induced alpha and beta suppression is less pronounced in PD participants compared to controls. TBS-DBS and cDBS reveal different patterns of movement-induced cortical activity patterns.

This observation was supported by the results of the cluster permutation test, which showed a high alpha and beta suppression for HCs, covering almost the entire cortex (Figure 3). In the cDBS and TBS-DBS conditions, significant clusters of movement-induced alpha and beta suppression emerged, though the suppression was not as strong as for the HCs and the clusters were topographically smaller. The subsequent qualitative, visual comparison of the topoplots (Figure 3) of the TBS-DBS and cDBS condition revealed different activity patterns. The suppression of low-beta band activity during the cDBS condition was primarily localized above the motor cortex, whereas the low-beta suppression in the TBS-DBS condition was additionally localized above the premotor cortex. Alpha activity suppression showed the opposite pattern. The alpha suppression in the TBS-DBS condition was predominantly localized above the motor cortex and in the cDBS condition above motor and premotor cortex.

**Figure 4.**
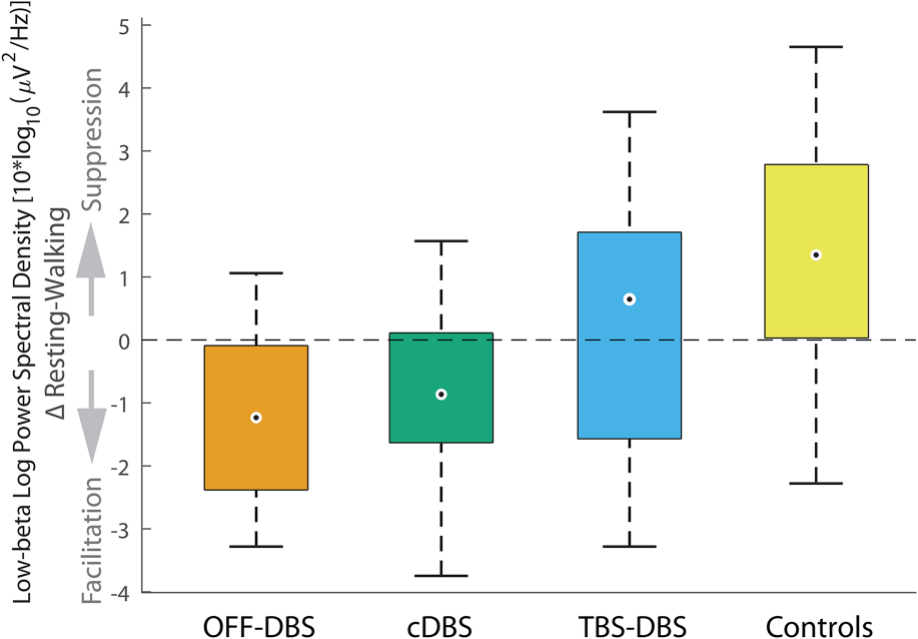
Movement-induced power modulation of low-beta activity in the frontal ROI. Boxplots for illustration of movement-induced EEG differences of frontal low-beta activity between all PD participants in OFF-DBS (orange), cDBS (green), TBS-DBS (blue) and HCs (yellow). Although there a qualitative preference of TBS-DBS in the extent of normalization of movement-induced low beta suppression compared to cDBS or OFF-DBS, it did not reach a significant level. The boxplots represent the 25th and 75th percentiles (box boundaries), while the whiskers indicate approximately 99.3% of the data (assuming a normal distribution). Outliers are marked as circles. Level of significance was set at 0.05.

The extent of low-beta suppression is also displayed in Figure 4, which illustrates the power difference between the “resting-like” and walking condition in the low-beta band in frontal ROI. Here, TBS-DBS showed a stronger low beta suppression than cDBS, although it was not statistically significant (TBS-DBS vs. cDBS *p* = 0.250; TBS-DBS vs. OFF-DBS *p* = 0.160; cDBS vs. OFF-DBS *p* = 0.570). EEG power amplitudes in the different frequency bands (theta, delta, alpha, low-beta, high-beta, gamma) were not significantly different between the assessed DBS conditions.

In an exploratory analysis, we also calculated a cluster permutation test between regular walking and freezing episodes where no significant clusters were found.

#### The TBS-preference and TBS-preference group did not show different cortical activity patterns

**Figure 5.**
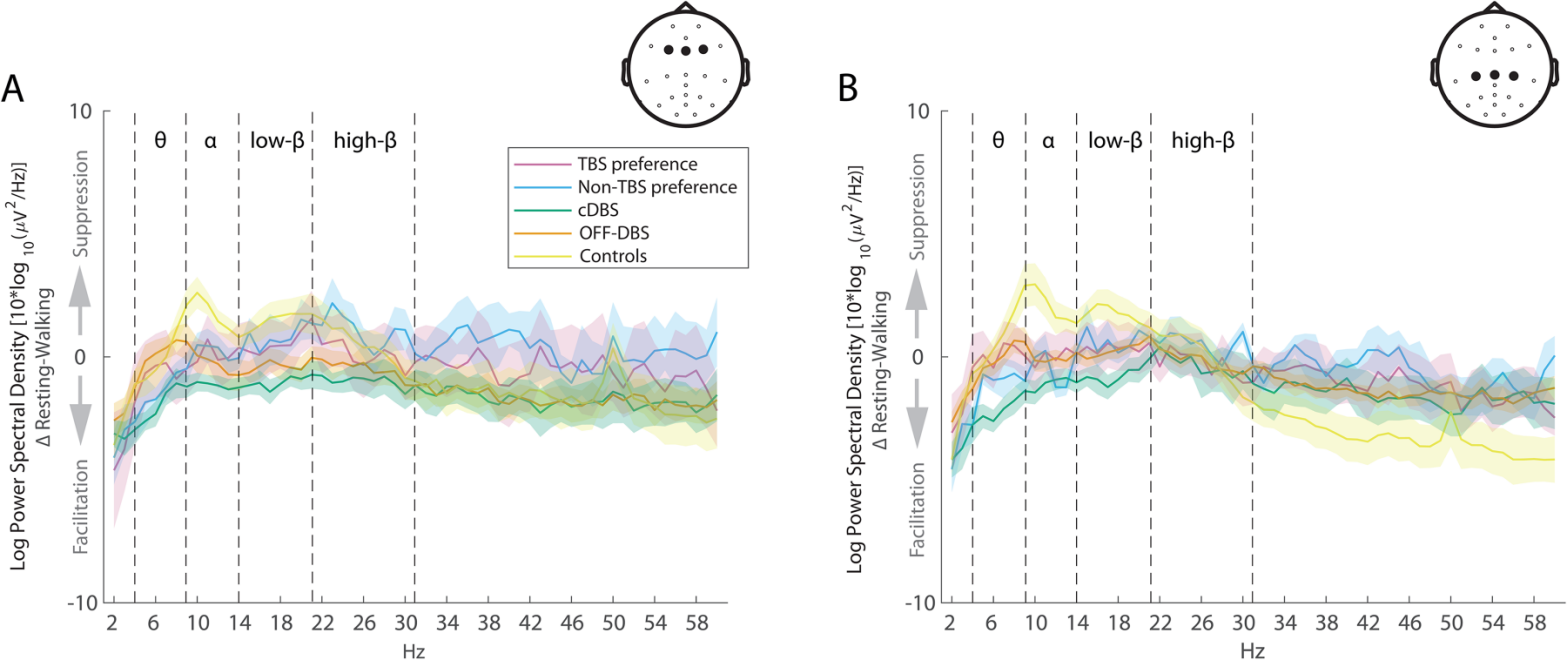
Movement-induced cortical activity changes in comparison between TBS preference and Non-TBS preference. Lineplots as in Figure 1 with the relative difference between resting-like and walking conditions in frontal ROI (A) and central ROI (B), in the TBS preference and Non-TBS preference subgroups. Shaded area represents standard error of the mean.

In a second step, we compared cortical activity patterns of the TBS-preference group and Non-TBS-preference group. The exploratory cluster permutation test of cortical activity changes, as measured by the EEG during the resting-like and walking condition, as well as the difference between resting-like and walking conditions in the TBS-preference group and the Non-TBS-preference group, did not reveal any significant clusters. Moreover, the line plots showing the activities of the TBS-preference group and Non-TBS-preference group (Figure 5), did not reveal any noticeable differences between the groups.

## Discussion

In this study, we found no superior effects of TBS-DBS compared to cDBS for the FoG-Course or the MDS-UPDRS III in the entirety of the group. However, within a specific subgroup of the TBS-preference group, the FoG-Course was significantly improved in TBS-DBS condition in comparison to OFF-DBS.

The MDS-UPDRS II score might be a possible predictor for becoming part of the TBS-preference group (although the beta weight was not significant, probably due to the small sample size). The TBS-preference group had a lower MDS-UPDRS II score than the Non-TBS-preference group, indicating a better overall health status in daily life situations with cDBS and medication. This finding could indicate that TBS-DBS cannot compensate for a poor effect of cDBS, e.g., due to misplaced DBS leads. In a generally working DBS setting, TBS-DBS might add the “icing on the cake” to improve the specific symptom complex gait disorder and FoG. The motor score of the observer-rated MDS-UPDRS III in MED OFF was not different between the TBS-preference group or Non-TBS-preference group, indicating that short-term, poor motor performance is not sufficient to predict TBS-repsonse on subjective impression of gait. Further predictive factors might be evaluated to elucidate potential TBS-preference in more detail.

Cortical network activity captured by mobile EEG revealed walking-induced alpha and beta suppression in both cDBS and TBS-DBS conditions, which was less accentuated compared to HC. This alpha and beta suppression was topographically overlapping, however, the beta suppression with TBS-DBS was more intense and not only limited to the motor cortex as during cDBS, but also included the premotor cortex. In the acute setting, these EEG changes were not accompanied by corresponding improvements of axial symptoms as measured by the MDS-UPDRS III. Since axial symptoms might change over days, the EEG might potentially point to advantageous long-term changes, which could not be proven in that acute experimental setting.

The most important limitation in the evaluation of patterned DBS algorithms is the assessment of short-term effects of TBS-DBS on gait within one day, since the effects of patterned DBS might evolve over time and axial symptoms typically respond over a longer time course. The 30-min wash-in time was an established waiting period in literature,^30^ but it might not be sufficient to assess changes of axial symptoms and gait. We chose to define the subgroups as the TBS-preference group and Non-TBS-preference group based on the patients’ self-assessment, rather than relying solely on objective parameters or the examiners’ evaluation. This approach intentionally prioritizes the patient's subjective perspective, which we believe is particularly relevant for future clinical applications. However, we acknowledge that there are alternative (and potentially more objective) classification options that could lead to different conclusions. Furthermore, we could have collected the patients’ assessments in more detail, for example using the seven point Clinical Global Impression Scale instead of a simple ranking between stimulation conditions. Despite various steps to reduce EEG artifacts due to DBS, gait or environmental confounders, it cannot be ruled out that the pre-processed EEG data may still contain artifacts. Additional constraints include the small sample size, especially in these analyses, where we had to exclude 4 PD participants with incomplete data sets due to severe symptom load in specific DBS conditions. Testing of PD participants on their regular medication could mask small differences or result in ceiling effects, but was done to re-create everyday framework conditions. One limitation is the unequal sex distribution restricting the generalizability of the results. This was due to specific recruitment issues with TBS-DBS available systems, strict inclusion criteria and the general problem of the worldwide uneven gender distribution of STN-DBS patients.^31–34^

Our EEG findings during gait and TBS-DBS integrate well with previous observations on spatially and spectrally segregated oscillatory activity changes in the pathophysiology of PD.^35^ At rest, PD patients show an excessive beta band activity in motor, premotor and frontal cortical areas,^36–38^ which is considered a hallmark of dopamine depletion.^39^ This excessive beta band activity correlates with PD motor symptoms such as bradykinesia and rigidity.^39^ This pathologically enhanced beta activity is synchronized throughout the cortex-basal ganglia network in PD with a distinct hierarchy of the cortex above the basal ganglia.^40^ Conventional STN-DBS suppresses the pathological increased beta activity and also reduces the beta coherence between motor cortex and basal ganglia, interpreted as a release of information flow from the basal ganglia output.^38,41^ In line with these results, our study reveals a significant beta suppression in the cDBS condition, which is slightly intensified with TBS-DBS, indicating an improved information flow between basal ganglia and motor cortex. There is a topographical difference of the beta distribution between the DBS conditions, TBS-DBS suppresses beta activity more widespread in the motor and premotor cortex compared to cDBS, potentially indicating a more effective release in different cortical areas of the pathologically beta activity.

Alpha band activity is associated with attention processes,^42^ which are highly relevant for the control of the Parkinsonian gait.^43^ In our study, movement-induced alpha-suppression was prominent in HCs, but was also found in PD participants with cDBS and TBS-DBS with slightly differential topographical patterns between both stimulation modes. This is in line with previous observations of stepping induced alpha power reduction in central clusters during STN-DBS and combined STN + nigral DBS.^29^ Alpha activity is present in a network including the STN, temporo-parietal cortex and brainstem regions.^40^ The functional role of alpha activity is proposed to facilitate relevant information by desynchronization and suppress irrelevant information through synchronization during action selection by increase of the signal to noise ratio.^42^ Thus TBS-DBS and cDBS might improve attentional information access through effective alpha desynchronization to improve gait.

TBS-DBS as a new algorithm for STN-DBS in PD is both unilaterally^7^ and bilaterally^8,44^ tolerable and safe. The spacing of stimulation trains and insertion of stimulation pauses with TBS-DBS might represent a double-edged sword for DBS application. On the one hand, there is evidence of re-emerging tremor during spaced thalamic deep brain stimulation depending on the length and timing of the stimulation pause.^45–47^ The finding of impaired, short-term efficacy in tremor suppression by non-continuous DBS was interpreted as the inability to mask burst-driver inputs to the thalamus.^47^ This might partially explain the slightly inferior short-term effect on objective gait parameters and the FoG-Course of TBS-DBS as a whole group compared to cDBS.

On the other hand, we assumed TBS-DBS to be potentially advantageous for gait due to the following reasons: (1) Previous studies suggested that nonregular patterns of STN-DBS with pauses were more effective in desynchronizing neural activity in PD and treatment-refractory obsessive-compulsive disorder compared to standard STN-DBS.^48^ Since in some studies, during or just before FoG, low STN beta oscillations are particularly increased,^49^ this exaggerated beta would then be sufficiently suppressed by spaced stimulation. (2) TBS-DBS with its temporal patterning might slowly, progressively increase its subthalamic efficacy in the long-term by beneficial neuroplastic effects from TBS-DBS.^50^ In the visuo-tectal systems^51^ or the hippocampus^52^ it was shown that bursting effects play a predominant role in determining the precision and specificity of synaptic plasticity. This was confirmed by providing a computational model of synaptic integration in the hippocampus that suggests the importance of the spacing effect and its potential role in late-phase plasticity.^53^ However, these effects only become apparent after a longer period of stimulation. For the exploration of the full effect strength, a long-term evaluation of TBS-DBS would be necessary, which has not been performed here in the randomised study of short-term effects. Sáenz-Farret et al. reported that a sub-group of PD participants responded well to TBS-DBS in the long-term evaluation, but another subgroup had no improvement of symptoms.^8^ This is in line with our observation that about half of the PD participants improved with TBS-DBS but not the other half. Therefore, an important next step would be to find predictors for the preference of patients to TBS-DBS and assess those patients in the long-term.

In conclusion, short-term bilateral TBS-DBS is safe and appears, at least in a subgroup with about half of the patients, to have a positive impact on gait disturbances and FoG, accompanied by specific cortical activity changes. At this point, it is too early to determine the potential role of TBS-DBS for trouble shooting of gait and FoG in clinical routine, further studies need to be performed to evaluate long-term effects in larger cohorts of patients.
